# Crystal structure of 1′,1′′-dimethyl-4′-(4-cholorophen­yl)di­spiro­[11*H*-indeno[1,2-*b*]quinoxaline-11,2′-pyrrolidine-3′,3′′-piperidin]-4′′-one

**DOI:** 10.1107/S2056989014027698

**Published:** 2015-01-03

**Authors:** R.A. Nagalakshmi, J. Suresh, K. Malathi, R. Ranjith Kumar, P. L. Nilantha Lakshman

**Affiliations:** a Department of Physics, The Madura College, Madurai 625 011, India; b Department of Organic Chemistry, School of Chemistry, Madurai Kamaraj University, Madurai 625 021, India; c Department of Food Science and Technology, University of Ruhuna, Mapalana, Kamburupitiya 81100, Sri Lanka

**Keywords:** crystal structure, quinoxaline derivative, intra­molecular C—H⋯N inter­action, C—H⋯π inter­action, Cl⋯Cl contact

## Abstract

In the title compound, C_30_H_27_ClN_4_O, the central pyrrolidine ring adopts an envelope conformation with the methyl­ene C atom being the flap. The quinoxaline and indane rings are each essentially planar, with r.m.s. deviations of 0.027 (1) and 0.0417 (1) Å, respectively. The pyrrolidine ring forms dihedral angles of 88.25 (1) and 83.76 (1)° with the quinoxaline and indane rings, respectively. A weak intra­molecular C—H⋯N inter­action is observed. In the crystal, C—H⋯π inter­actions lead to supra­molecular chains along [101] that assemble in the *ac* plane. Connections along the *b* axis are of the type Cl⋯Cl [3.6538 (16) Å].

## Related literature   

For the importance of quinoxaline derivatives, see: Abasolo *et al.* (1987 ▸); Kleim *et al.* (1995 ▸); Dailey *et al.* (2001 ▸); Rodrigo *et al.* (2002 ▸); Seitz *et al.* (2002 ▸). For a related structure, see: Selvanayagam *et al.* (2011 ▸).
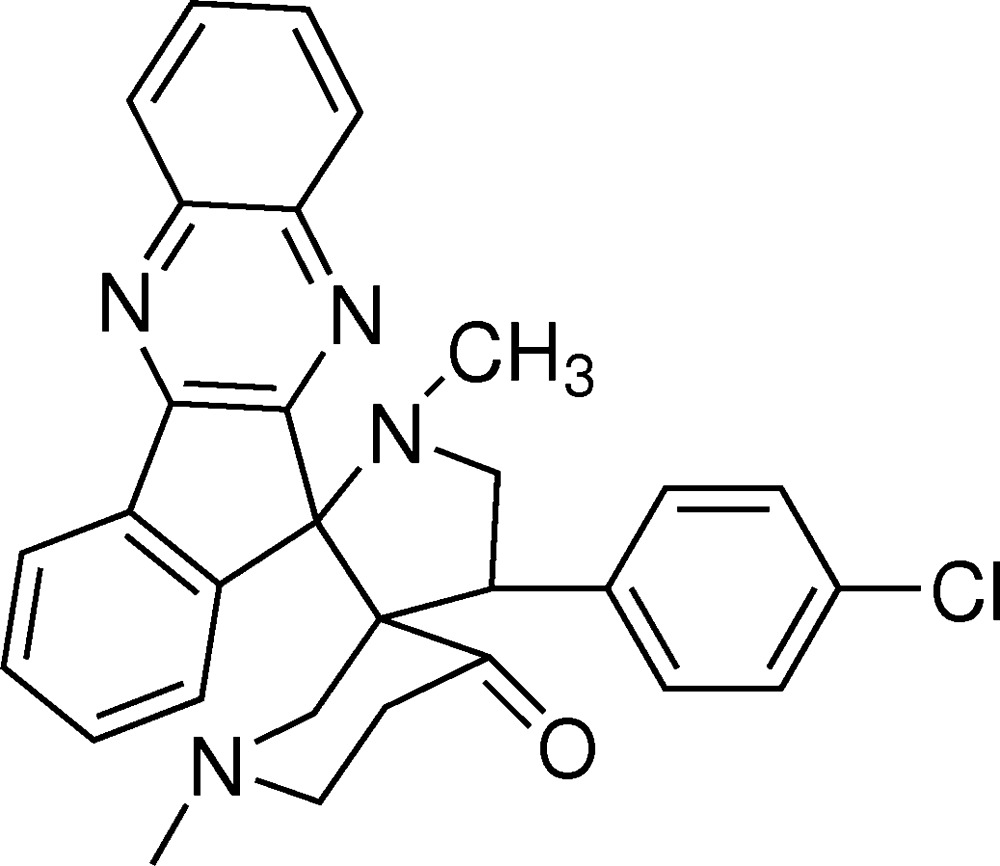


## Experimental   

### Crystal data  

C_30_H_27_ClN_4_O
*M* *_r_* = 495.00
Monoclinic, 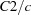

*a* = 22.4220 (13) Å
*b* = 14.3498 (9) Å
*c* = 17.2811 (9) Å
β = 116.847 (2)°
*V* = 4960.9 (5) Å^3^
*Z* = 8
Mo *K*α radiation
μ = 0.19 mm^−1^
*T* = 293 K
0.21 × 0.19 × 0.18 mm

### Data collection  

Bruker Kappa APEXII diffractometer
Absorption correction: multi-scan (*SADABS*; Sheldrick, 1996 ▸) *T*_min_ = 0.967, *T*_max_ = 0.974
18093 measured reflections
3791 independent reflections
2758 reflections with *I* > 2σ(*I*)
*R*_int_ = 0.032

### Refinement  

*R*[*F* ^2^ > 2σ(*F* ^2^)] = 0.041
*wR*(*F* ^2^) = 0.116
*S* = 1.05
3791 reflections
325 parameters
H-atom parameters constrained
Δρ_max_ = 0.22 e Å^−3^
Δρ_min_ = −0.26 e Å^−3^

### 

Data collection: *APEX2* (Bruker, 2004 ▸); cell refinement: *SAINT* (Bruker, 2004 ▸); data reduction: *SAINT*; program(s) used to solve structure: *SHELXS97* (Sheldrick, 2008 ▸); program(s) used to refine structure: *SHELXL2014* (Sheldrick, 2008 ▸); molecular graphics: *PLATON* (Spek, 2009 ▸); software used to prepare material for publication: *SHELXL2014*.

## Supplementary Material

Crystal structure: contains datablock(s) global, I. DOI: 10.1107/S2056989014027698/zq2230sup1.cif

Structure factors: contains datablock(s) I. DOI: 10.1107/S2056989014027698/zq2230Isup2.hkl

Supporting information file. DOI: 10.1107/S2056989014027698/zq2230Isup3.cml

**Fig. 1.**
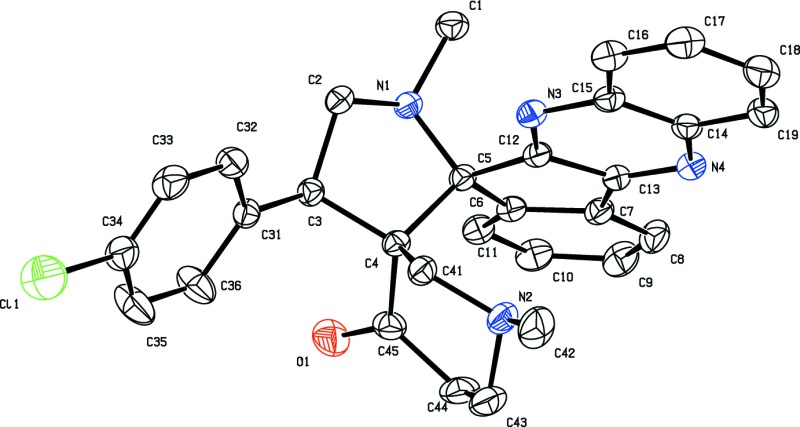
The molecular structure of the title compound, showing 30% probability displacement ellipsoids and the atom-numbering scheme. H-atoms are omitted for clarity.

. DOI: 10.1107/S2056989014027698/zq2230fig1.tif
The mol­ecular structure of the title compound, showing 30% probability displacement ellipsoids and the atom-numbering scheme. H-atoms are omitted for clarity.

**Fig. 2.**
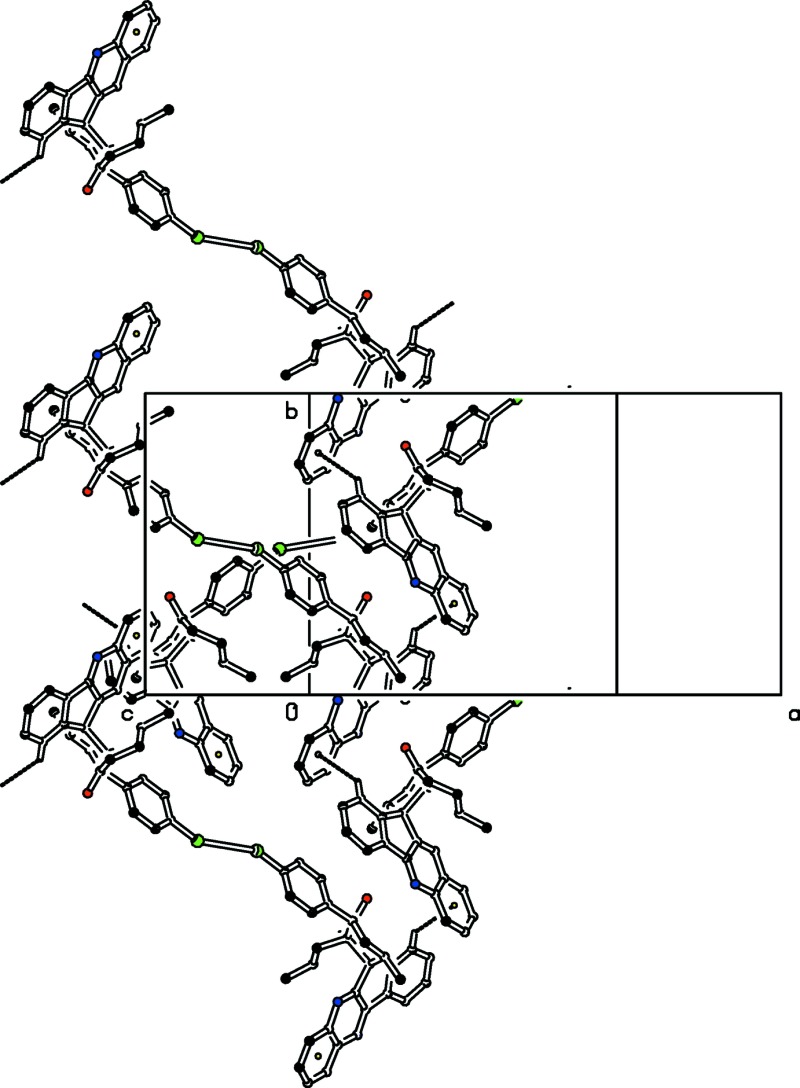
The partial packing diagram showing C—H···π interactions as dashed lines. All H-atoms are omitted for clarity except for the H atom involved in the intermolecular interaction.

. DOI: 10.1107/S2056989014027698/zq2230fig2.tif
The partial packing diagram showing C—H⋯π inter­actions as dashed lines. All H-atoms are omitted for clarity except for the H atom involved in the inter­molecular inter­action.

CCDC reference: 1040368

Additional supporting information:  crystallographic information; 3D view; checkCIF report

## Figures and Tables

**Table 1:** Hydrogen-bond geometry (, ) *Cg*1 is the centroid of the C14C19 ring.

*D*H*A*	*D*H	H*A*	*D* *A*	*D*H*A*
C41H41*A*N3	0.97	2.39	2.988(3)	120
C11H11*Cg*1^i^	0.93	2.89	3.655(2)	140

Symmetry code: (i) 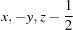.

## supplementary crystallographic information

### S1. Chemical context

Quinoxaline derivatives are an important class of benzoheterocycles. They have found applications as anti-cancer, anti-viral, and anti-bacterial agents (Seitz *et al.*, 2002), and dyes (Dailey *et al.*, 2001). Quinoxaline derivatives were found to exhibit anti-microbial (Kleim *et al.* 1995), anti­tumor (Abasolo *et al.*, 1987), and anti-tuberculous activity (Rodrigo *et al.*, 2002). Our inter­est in preparing pharmacologically active quinoxaline derivatives led us to the title compound, and we have undertaken X-ray crystal structure determination in order to establish its conformation.

### S2. Structural commentary

In the title compound (Fig. 1), C_30_H_27_ClN_4_O, the central pyrrolidine ring is an envelope on C2 with the asymmetry parameters ΔC_s_(C2) = 7.6 (2) Å and puckering parameters q_2_ = 0.433 (2) Å and φ_2_ = 31.2 (3)°. The pyrrolidine ring is almost equatorial with the quinoxaline and indane rings making dihedral angles of 88.34 (1)° and 83.71 (1)°, respectively. The quinoxaline ring system (C12—C17/N3,N4) is planar, with r.m.s. deviation of 0.027 (1) Å. The indane group is also planar with r.m.s.deviation 0.0417 (1)°. The dihedral angle between the mean planes of the fused quinoxaline and the indane groups is 8.39 (1) °, which indicates that the fused rings are slightly twisted about the C12—C13 bond. The C—C bond lengths in the pyrrolidine ring in particular, at two spiro junctions (C3—C4 = 1.558 (3) Å and C4—C5 = 1.605 (3) Å) are somewhat longer than the normal values (C—C = 1.54 Å), as found in a similar structure (Selvanayagam *et al.*, 2011). This may be due to the the steric inter­actions of the bulky substituents at atoms C4 and C5 of the pyrrolidine ring. The short contact H32 ··· H2b of 2.18 Å results in substantial widening of the bond angle C3—C31—C32 to 123.67 (1) °. The sum of the bond angles around N1 (339.87 (1)°) and N2 (330.97 (1)°) indicate that the atoms N1 and N2 exhibit a pyramidal geometry. The six membered ring N2/C41—C45 exhibits a twisted chair conformation, as indicated by the assymetrical parameters ΔC_s_(N2) = 7.4 (2)°, ΔC_s_(C45) = 7.4 (2) ° and with the puckering parameters Q = 0.559 (3) Å, θ = 17.4 (3)° and Φ = 24.6 (9)°. The torsion angle C4—C41—N2—C42 is -167.50 ° corresponds to an anti­periplanar conformation.

A weak intra-molecular C—H···N inter­action is observed (Table 1). In the crystal, C—H···π inter­actions lead to supra­molecular chains along [101] that assemble in the ac plane. Connections along the *b* axis are of the type Cl···Cl (3.6538 (16) Å).

### S3. Synthesis and crystallization

A mixture of 1-methyl-3-[E-(4-chloro­phenyl)­methyl­idene]tetra­hydro-2(1H)- pyridinone (1 mmol), ninhydrin (1 mmol), o-phenyl­enedi­amine (1 mmol) and sarcosine (1 mmol) in methanol was refluxed for 3-4 h. After completion of the reaction as indicated by TLC the reaction mixture was poured into cold water. The solid precipitate obtained was filtered and dried. The product was purified by colum chromatography using petroleum ether:ethyl­acetate mixture (90:10 V/V). Suitable crystals for the single crystal-X-ray studies were obtained by recrystalizing the product from methanol. Yield: 42%, Melting point: 450-452 K.

### S4. Refinement

All the H atoms were placed at calculated positions and allowed to ride on their carrier atoms with C—H = 0.93–0.98 Å, and with *U*_iso_ = 1.2*U*_eq_(C) for CH_2_ and CH groups, and *U*_iso_ = 1.5*U*_eq_(C) for CH_3_ groups. The (-1 1 1), (1 1 0) reflections were affected by the beam-stop and were removed from the final refinement. The best crystal investigated was still of poor quality and very weakly diffracting, with no usable data obtanied above θ = 23.8°. Nonetheless, the structure was solved readily and refined to give acceptable uncertainties on the metrical data.

### Crystal data

**Table:**

C_30_H_27_ClN_4_O	*F*(000) = 2080
*M**_r_* = 495.00	*D*_x_ = 1.326 Mg m^−^^3^
Monoclinic, *C*2/*c*	Mo *K*α radiation, λ = 0.71073 Å
*a* = 22.4220 (13) Å	Cell parameters from 2000 reflections
*b* = 14.3498 (9) Å	θ = 2–31°
*c* = 17.2811 (9) Å	µ = 0.19 mm^−^^1^
β = 116.847 (2)°	*T* = 293 K
*V* = 4960.9 (5) Å^3^	Block, colourless
*Z* = 8	0.21 × 0.19 × 0.18 mm

### Data collection

**Table:**

Bruker Kappa APEXII diffractometer	2758 reflections with *I* > 2σ(*I*)
Radiation source: fine-focus sealed tube	*R*_int_ = 0.032
ω and φ scans	θ_max_ = 23.8°, θ_min_ = 2.0°
Absorption correction: multi-scan (*SADABS*; Sheldrick, 1996)	*h* = −24→25
*T*_min_ = 0.967, *T*_max_ = 0.974	*k* = −16→16
18093 measured reflections	*l* = −19→13
3791 independent reflections	

### Refinement

**Table:**

Refinement on *F*^2^	0 restraints
Least-squares matrix: full	Hydrogen site location: inferred from neighbouring sites
*R*[*F*^2^ > 2σ(*F*^2^)] = 0.041	H-atom parameters constrained
*wR*(*F*^2^) = 0.116	*w* = 1/[σ^2^(*F*_o_^2^) + (0.052*P*)^2^ + 2.7359*P*] where *P* = (*F*_o_^2^ + 2*F*_c_^2^)/3
*S* = 1.05	(Δ/σ)_max_ < 0.001
3791 reflections	Δρ_max_ = 0.22 e Å^−^^3^
325 parameters	Δρ_min_ = −0.26 e Å^−^^3^

### Special details

**Table:**

Geometry. All e.s.d.'s (except the e.s.d. in the dihedral angle between two l.s. planes) are estimated using the full covariance matrix. The cell e.s.d.'s are taken into account individually in the estimation of e.s.d.'s in distances, angles and torsion angles; correlations between e.s.d.'s in cell parameters are only used when they are defined by crystal symmetry. An approximate (isotropic) treatment of cell e.s.d.'s is used for estimating e.s.d.'s involving l.s. planes.

### Fractional atomic coordinates and isotropic or equivalent isotropic displacement parameters (Å^2^)

**Table:**

	*x*	*y*	*z*	*U*_iso_*/*U*_eq_	
C1	0.30822 (11)	0.05687 (16)	0.33108 (14)	0.0608 (6)	
H1A	0.3191	0.0297	0.2883	0.091*	
H1B	0.3481	0.0807	0.3781	0.091*	
H1C	0.2888	0.0103	0.3526	0.091*	
C2	0.23954 (11)	0.18035 (15)	0.34879 (13)	0.0523 (5)	
H2A	0.2767	0.2104	0.3965	0.063*	
H2B	0.2174	0.1385	0.3717	0.063*	
C3	0.19136 (10)	0.25089 (14)	0.28740 (12)	0.0459 (5)	
H3	0.2184	0.2962	0.2746	0.055*	
C4	0.15164 (10)	0.19405 (14)	0.20256 (12)	0.0457 (5)	
C5	0.20122 (9)	0.10932 (14)	0.21204 (12)	0.0439 (5)	
C6	0.22053 (10)	0.09623 (14)	0.13862 (12)	0.0466 (5)	
C7	0.19965 (10)	0.01074 (15)	0.09751 (12)	0.0492 (5)	
C8	0.21358 (12)	−0.01455 (18)	0.03003 (14)	0.0630 (6)	
H8	0.1994	−0.0717	0.0024	0.076*	
C9	0.24862 (13)	0.0464 (2)	0.00475 (16)	0.0714 (7)	
H9	0.2575	0.0308	−0.0413	0.086*	
C10	0.27083 (12)	0.13009 (18)	0.04655 (14)	0.0650 (6)	
H10	0.2953	0.1699	0.0292	0.078*	
C11	0.25718 (10)	0.15583 (16)	0.11420 (13)	0.0556 (6)	
H11	0.2725	0.2124	0.1426	0.067*	
C12	0.17122 (9)	0.01365 (14)	0.21185 (12)	0.0447 (5)	
C13	0.16878 (10)	−0.04160 (14)	0.14202 (12)	0.0462 (5)	
C14	0.12375 (10)	−0.15970 (15)	0.18401 (13)	0.0513 (5)	
C15	0.12990 (10)	−0.10743 (15)	0.25619 (13)	0.0506 (5)	
C16	0.11002 (11)	−0.14672 (17)	0.31511 (15)	0.0628 (6)	
H16	0.1147	−0.1130	0.3635	0.075*	
C17	0.08385 (12)	−0.23439 (19)	0.30153 (17)	0.0719 (7)	
H17	0.0710	−0.2604	0.3410	0.086*	
C18	0.07626 (12)	−0.28531 (18)	0.22925 (18)	0.0737 (7)	
H18	0.0576	−0.3446	0.2202	0.088*	
C19	0.09589 (11)	−0.24923 (16)	0.17162 (15)	0.0623 (6)	
H19	0.0908	−0.2842	0.1237	0.075*	
C31	0.15093 (10)	0.30600 (14)	0.32168 (13)	0.0483 (5)	
C32	0.14179 (12)	0.27914 (17)	0.39179 (14)	0.0640 (6)	
H32	0.1598	0.2228	0.4188	0.077*	
C33	0.10678 (13)	0.3330 (2)	0.42345 (15)	0.0742 (7)	
H33	0.1012	0.3126	0.4709	0.089*	
C34	0.08049 (13)	0.4154 (2)	0.38554 (17)	0.0752 (7)	
C35	0.08847 (17)	0.4439 (2)	0.3162 (2)	0.1013 (10)	
H35	0.0704	0.5005	0.2898	0.122*	
C36	0.12311 (15)	0.38969 (18)	0.28474 (18)	0.0839 (8)	
H36	0.1278	0.4104	0.2368	0.101*	
C41	0.08396 (10)	0.16135 (16)	0.19371 (13)	0.0539 (5)	
H41A	0.0908	0.1229	0.2432	0.065*	
H41B	0.0576	0.2151	0.1934	0.065*	
C42	−0.00945 (13)	0.0602 (2)	0.1155 (2)	0.0974 (10)	
H42A	0.0060	0.0193	0.1647	0.146*	
H42B	−0.0396	0.1053	0.1193	0.146*	
H42C	−0.0322	0.0244	0.0632	0.146*	
C43	0.02670 (14)	0.1710 (2)	0.04037 (16)	0.0857 (9)	
H43A	−0.0012	0.2199	0.0455	0.103*	
H43B	0.0004	0.1369	−0.0128	0.103*	
C44	0.08668 (14)	0.2140 (2)	0.03617 (14)	0.0824 (8)	
H44A	0.1079	0.1672	0.0164	0.099*	
H44B	0.0716	0.2639	−0.0063	0.099*	
C45	0.13753 (13)	0.25230 (18)	0.12140 (14)	0.0612 (6)	
N1	0.26100 (8)	0.13210 (12)	0.29228 (10)	0.0486 (4)	
N2	0.04763 (9)	0.10806 (14)	0.11417 (12)	0.0668 (5)	
N3	0.15437 (8)	−0.01733 (12)	0.27018 (10)	0.0499 (4)	
N4	0.14498 (8)	−0.12662 (12)	0.12568 (11)	0.0532 (5)	
O1	0.16569 (11)	0.32522 (13)	0.12566 (11)	0.0874 (6)	
Cl1	0.03708 (5)	0.48362 (8)	0.42608 (6)	0.1348 (4)	

### Atomic displacement parameters (Å^2^)

**Table:**

	*U*^11^	*U*^22^	*U*^33^	*U*^12^	*U*^13^	*U*^23^
C1	0.0526 (13)	0.0683 (16)	0.0540 (13)	0.0102 (11)	0.0175 (11)	0.0012 (11)
C2	0.0550 (13)	0.0562 (13)	0.0397 (11)	0.0015 (10)	0.0160 (10)	−0.0042 (10)
C3	0.0513 (12)	0.0446 (12)	0.0423 (11)	−0.0033 (9)	0.0215 (10)	−0.0010 (9)
C4	0.0502 (12)	0.0497 (13)	0.0360 (10)	0.0035 (10)	0.0185 (9)	0.0018 (9)
C5	0.0458 (11)	0.0479 (12)	0.0362 (10)	0.0004 (9)	0.0168 (9)	−0.0014 (9)
C6	0.0451 (11)	0.0539 (13)	0.0382 (11)	0.0029 (10)	0.0164 (9)	0.0037 (10)
C7	0.0484 (12)	0.0581 (14)	0.0380 (11)	0.0033 (10)	0.0167 (10)	−0.0017 (10)
C8	0.0732 (16)	0.0713 (16)	0.0464 (13)	−0.0009 (13)	0.0287 (12)	−0.0120 (11)
C9	0.0785 (17)	0.094 (2)	0.0515 (14)	0.0000 (15)	0.0383 (13)	−0.0059 (14)
C10	0.0638 (15)	0.0843 (19)	0.0534 (14)	−0.0014 (13)	0.0322 (12)	0.0078 (13)
C11	0.0566 (13)	0.0617 (15)	0.0488 (12)	−0.0004 (11)	0.0241 (11)	0.0025 (11)
C12	0.0424 (11)	0.0516 (13)	0.0371 (11)	0.0039 (9)	0.0153 (9)	0.0017 (9)
C13	0.0440 (11)	0.0480 (13)	0.0402 (11)	0.0032 (10)	0.0133 (9)	0.0003 (10)
C14	0.0428 (11)	0.0502 (14)	0.0500 (12)	0.0033 (10)	0.0112 (10)	0.0067 (10)
C15	0.0408 (11)	0.0565 (14)	0.0470 (12)	−0.0002 (10)	0.0133 (10)	0.0055 (10)
C16	0.0532 (13)	0.0751 (17)	0.0574 (14)	−0.0066 (12)	0.0226 (11)	0.0095 (12)
C17	0.0577 (15)	0.0799 (19)	0.0707 (17)	−0.0117 (13)	0.0223 (13)	0.0178 (14)
C18	0.0583 (15)	0.0604 (16)	0.0869 (19)	−0.0086 (12)	0.0190 (14)	0.0120 (15)
C19	0.0554 (14)	0.0506 (14)	0.0665 (15)	−0.0019 (11)	0.0149 (12)	0.0030 (11)
C31	0.0535 (12)	0.0455 (13)	0.0454 (12)	−0.0062 (10)	0.0218 (10)	−0.0037 (9)
C32	0.0822 (17)	0.0618 (15)	0.0521 (13)	0.0109 (13)	0.0339 (13)	0.0044 (11)
C33	0.0803 (17)	0.098 (2)	0.0518 (14)	0.0089 (16)	0.0364 (14)	−0.0049 (14)
C34	0.0730 (17)	0.085 (2)	0.0645 (16)	0.0170 (15)	0.0286 (14)	−0.0128 (14)
C35	0.143 (3)	0.0686 (19)	0.120 (3)	0.0444 (19)	0.084 (2)	0.0252 (18)
C36	0.122 (2)	0.0625 (17)	0.096 (2)	0.0280 (16)	0.0741 (19)	0.0268 (15)
C41	0.0477 (12)	0.0577 (14)	0.0505 (12)	0.0040 (10)	0.0170 (10)	−0.0077 (10)
C42	0.0565 (16)	0.100 (2)	0.111 (2)	−0.0149 (15)	0.0164 (16)	−0.0301 (18)
C43	0.0708 (18)	0.103 (2)	0.0537 (15)	0.0192 (16)	0.0022 (14)	−0.0080 (15)
C44	0.090 (2)	0.103 (2)	0.0409 (13)	0.0323 (17)	0.0177 (14)	0.0129 (13)
C45	0.0720 (16)	0.0648 (16)	0.0493 (13)	0.0192 (13)	0.0295 (12)	0.0092 (12)
N1	0.0462 (10)	0.0531 (11)	0.0410 (9)	0.0039 (8)	0.0148 (8)	−0.0023 (8)
N2	0.0516 (11)	0.0734 (14)	0.0582 (12)	0.0014 (10)	0.0096 (10)	−0.0157 (10)
N3	0.0496 (10)	0.0564 (12)	0.0424 (10)	−0.0024 (8)	0.0198 (8)	0.0016 (8)
N4	0.0516 (10)	0.0521 (11)	0.0475 (10)	0.0005 (9)	0.0151 (9)	−0.0010 (9)
O1	0.1327 (17)	0.0656 (12)	0.0729 (12)	0.0032 (12)	0.0542 (12)	0.0171 (10)
Cl1	0.1370 (8)	0.1672 (9)	0.1083 (7)	0.0676 (7)	0.0626 (6)	−0.0185 (6)

### Geometric parameters (Å, º)

**Table:**

C1—N1	1.447 (3)	C15—C16	1.401 (3)
C1—H1A	0.9600	C16—C17	1.363 (3)
C1—H1B	0.9600	C16—H16	0.9300
C1—H1C	0.9600	C17—C18	1.390 (4)
C2—N1	1.445 (2)	C17—H17	0.9300
C2—C3	1.511 (3)	C18—C19	1.360 (3)
C2—H2A	0.9700	C18—H18	0.9300
C2—H2B	0.9700	C19—H19	0.9300
C3—C31	1.511 (3)	C31—C36	1.371 (3)
C3—C4	1.558 (3)	C31—C32	1.372 (3)
C3—H3	0.9800	C32—C33	1.379 (3)
C4—C41	1.529 (3)	C32—H32	0.9300
C4—C45	1.538 (3)	C33—C34	1.352 (4)
C4—C5	1.605 (3)	C33—H33	0.9300
C5—N1	1.466 (2)	C34—C35	1.352 (4)
C5—C6	1.526 (3)	C34—Cl1	1.736 (2)
C5—C12	1.528 (3)	C35—C36	1.374 (4)
C6—C11	1.378 (3)	C35—H35	0.9300
C6—C7	1.389 (3)	C36—H36	0.9300
C7—C8	1.385 (3)	C41—N2	1.458 (3)
C7—C13	1.455 (3)	C41—H41A	0.9700
C8—C9	1.372 (3)	C41—H41B	0.9700
C8—H8	0.9300	C42—N2	1.462 (3)
C9—C10	1.374 (3)	C42—H42A	0.9600
C9—H9	0.9300	C42—H42B	0.9600
C10—C11	1.386 (3)	C42—H42C	0.9600
C10—H10	0.9300	C43—N2	1.457 (3)
C11—H11	0.9300	C43—C44	1.511 (4)
C12—N3	1.304 (2)	C43—H43A	0.9700
C12—C13	1.424 (3)	C43—H43B	0.9700
C13—N4	1.310 (3)	C44—C45	1.501 (4)
C14—N4	1.379 (3)	C44—H44A	0.9700
C14—C19	1.402 (3)	C44—H44B	0.9700
C14—C15	1.408 (3)	C45—O1	1.207 (3)
C15—N3	1.382 (3)		
			
N1—C1—H1A	109.5	C16—C17—C18	120.6 (2)
N1—C1—H1B	109.5	C16—C17—H17	119.7
H1A—C1—H1B	109.5	C18—C17—H17	119.7
N1—C1—H1C	109.5	C19—C18—C17	120.6 (2)
H1A—C1—H1C	109.5	C19—C18—H18	119.7
H1B—C1—H1C	109.5	C17—C18—H18	119.7
N1—C2—C3	101.37 (15)	C18—C19—C14	120.4 (2)
N1—C2—H2A	111.5	C18—C19—H19	119.8
C3—C2—H2A	111.5	C14—C19—H19	119.8
N1—C2—H2B	111.5	C36—C31—C32	116.1 (2)
C3—C2—H2B	111.5	C36—C31—C3	120.23 (19)
H2A—C2—H2B	109.3	C32—C31—C3	123.67 (19)
C2—C3—C31	116.15 (16)	C31—C32—C33	122.1 (2)
C2—C3—C4	103.51 (15)	C31—C32—H32	118.9
C31—C3—C4	116.98 (16)	C33—C32—H32	118.9
C2—C3—H3	106.5	C34—C33—C32	120.0 (2)
C31—C3—H3	106.5	C34—C33—H33	120.0
C4—C3—H3	106.5	C32—C33—H33	120.0
C41—C4—C45	106.83 (17)	C35—C34—C33	119.4 (2)
C41—C4—C3	112.00 (15)	C35—C34—Cl1	120.6 (2)
C45—C4—C3	111.62 (17)	C33—C34—Cl1	119.9 (2)
C41—C4—C5	112.83 (16)	C34—C35—C36	120.3 (3)
C45—C4—C5	110.64 (15)	C34—C35—H35	119.9
C3—C4—C5	103.02 (15)	C36—C35—H35	119.9
N1—C5—C6	109.41 (15)	C31—C36—C35	122.1 (2)
N1—C5—C12	114.58 (15)	C31—C36—H36	118.9
C6—C5—C12	100.23 (15)	C35—C36—H36	118.9
N1—C5—C4	102.88 (14)	N2—C41—C4	111.24 (16)
C6—C5—C4	116.86 (15)	N2—C41—H41A	109.4
C12—C5—C4	113.36 (15)	C4—C41—H41A	109.4
C11—C6—C7	120.10 (18)	N2—C41—H41B	109.4
C11—C6—C5	127.65 (19)	C4—C41—H41B	109.4
C7—C6—C5	112.17 (17)	H41A—C41—H41B	108.0
C8—C7—C6	120.7 (2)	N2—C42—H42A	109.5
C8—C7—C13	130.7 (2)	N2—C42—H42B	109.5
C6—C7—C13	108.46 (17)	H42A—C42—H42B	109.5
C9—C8—C7	118.7 (2)	N2—C42—H42C	109.5
C9—C8—H8	120.6	H42A—C42—H42C	109.5
C7—C8—H8	120.6	H42B—C42—H42C	109.5
C8—C9—C10	120.9 (2)	N2—C43—C44	110.7 (2)
C8—C9—H9	119.6	N2—C43—H43A	109.5
C10—C9—H9	119.6	C44—C43—H43A	109.5
C9—C10—C11	120.7 (2)	N2—C43—H43B	109.5
C9—C10—H10	119.6	C44—C43—H43B	109.5
C11—C10—H10	119.6	H43A—C43—H43B	108.1
C6—C11—C10	118.9 (2)	C45—C44—C43	113.6 (2)
C6—C11—H11	120.6	C45—C44—H44A	108.8
C10—C11—H11	120.6	C43—C44—H44A	108.8
N3—C12—C13	123.48 (19)	C45—C44—H44B	108.8
N3—C12—C5	125.84 (17)	C43—C44—H44B	108.8
C13—C12—C5	110.51 (16)	H44A—C44—H44B	107.7
N4—C13—C12	123.85 (18)	O1—C45—C44	121.4 (2)
N4—C13—C7	127.70 (18)	O1—C45—C4	121.9 (2)
C12—C13—C7	108.38 (18)	C44—C45—C4	116.6 (2)
N4—C14—C19	118.7 (2)	C2—N1—C1	116.20 (16)
N4—C14—C15	122.43 (19)	C2—N1—C5	107.75 (15)
C19—C14—C15	118.8 (2)	C1—N1—C5	115.92 (16)
N3—C15—C16	118.7 (2)	C43—N2—C41	108.79 (19)
N3—C15—C14	121.71 (18)	C43—N2—C42	111.5 (2)
C16—C15—C14	119.6 (2)	C41—N2—C42	110.65 (19)
C17—C16—C15	120.0 (2)	C12—N3—C15	114.47 (17)
C17—C16—H16	120.0	C13—N4—C14	113.86 (17)
C15—C16—H16	120.0		
			
N1—C2—C3—C31	171.55 (16)	C14—C15—C16—C17	−1.1 (3)
N1—C2—C3—C4	41.92 (19)	C15—C16—C17—C18	−0.4 (4)
C2—C3—C4—C41	98.92 (19)	C16—C17—C18—C19	1.2 (4)
C31—C3—C4—C41	−30.2 (2)	C17—C18—C19—C14	−0.4 (4)
C2—C3—C4—C45	−141.35 (17)	N4—C14—C19—C18	178.0 (2)
C31—C3—C4—C45	89.5 (2)	C15—C14—C19—C18	−1.1 (3)
C2—C3—C4—C5	−22.61 (18)	C2—C3—C31—C36	158.8 (2)
C31—C3—C4—C5	−151.73 (16)	C4—C3—C31—C36	−78.4 (3)
C41—C4—C5—N1	−125.30 (16)	C2—C3—C31—C32	−18.6 (3)
C45—C4—C5—N1	115.08 (18)	C4—C3—C31—C32	104.2 (2)
C3—C4—C5—N1	−4.34 (18)	C36—C31—C32—C33	−0.1 (4)
C41—C4—C5—C6	114.83 (18)	C3—C31—C32—C33	177.4 (2)
C45—C4—C5—C6	−4.8 (2)	C31—C32—C33—C34	−0.4 (4)
C3—C4—C5—C6	−124.21 (17)	C32—C33—C34—C35	0.5 (4)
C41—C4—C5—C12	−1.0 (2)	C32—C33—C34—Cl1	−179.4 (2)
C45—C4—C5—C12	−120.61 (19)	C33—C34—C35—C36	−0.1 (5)
C3—C4—C5—C12	119.97 (16)	Cl1—C34—C35—C36	179.8 (3)
N1—C5—C6—C11	−51.1 (3)	C32—C31—C36—C35	0.4 (4)
C12—C5—C6—C11	−171.90 (19)	C3—C31—C36—C35	−177.1 (3)
C4—C5—C6—C11	65.2 (3)	C34—C35—C36—C31	−0.3 (5)
N1—C5—C6—C7	125.62 (18)	C45—C4—C41—N2	57.7 (2)
C12—C5—C6—C7	4.8 (2)	C3—C4—C41—N2	−179.81 (16)
C4—C5—C6—C7	−118.06 (19)	C5—C4—C41—N2	−64.1 (2)
C11—C6—C7—C8	−1.9 (3)	N2—C43—C44—C45	−47.8 (3)
C5—C6—C7—C8	−178.92 (18)	C43—C44—C45—O1	−139.5 (3)
C11—C6—C7—C13	173.82 (18)	C43—C44—C45—C4	40.4 (3)
C5—C6—C7—C13	−3.2 (2)	C41—C4—C45—O1	136.3 (2)
C6—C7—C8—C9	0.2 (3)	C3—C4—C45—O1	13.6 (3)
C13—C7—C8—C9	−174.4 (2)	C5—C4—C45—O1	−100.5 (2)
C7—C8—C9—C10	1.4 (4)	C41—C4—C45—C44	−43.6 (2)
C8—C9—C10—C11	−1.3 (4)	C3—C4—C45—C44	−166.30 (19)
C7—C6—C11—C10	2.0 (3)	C5—C4—C45—C44	79.6 (2)
C5—C6—C11—C10	178.47 (19)	C3—C2—N1—C1	−179.25 (17)
C9—C10—C11—C6	−0.4 (3)	C3—C2—N1—C5	−47.3 (2)
N1—C5—C12—N3	53.5 (3)	C6—C5—N1—C2	156.77 (16)
C6—C5—C12—N3	170.50 (18)	C12—C5—N1—C2	−91.62 (19)
C4—C5—C12—N3	−64.2 (2)	C4—C5—N1—C2	31.88 (19)
N1—C5—C12—C13	−121.80 (17)	C6—C5—N1—C1	−71.1 (2)
C6—C5—C12—C13	−4.8 (2)	C12—C5—N1—C1	40.5 (2)
C4—C5—C12—C13	120.51 (17)	C4—C5—N1—C1	164.01 (16)
N3—C12—C13—N4	5.1 (3)	C44—C43—N2—C41	62.2 (3)
C5—C12—C13—N4	−179.49 (18)	C44—C43—N2—C42	−175.5 (2)
N3—C12—C13—C7	−172.11 (18)	C4—C41—N2—C43	−69.7 (2)
C5—C12—C13—C7	3.3 (2)	C4—C41—N2—C42	167.5 (2)
C8—C7—C13—N4	−2.0 (4)	C13—C12—N3—C15	−3.6 (3)
C6—C7—C13—N4	−177.16 (19)	C5—C12—N3—C15	−178.36 (17)
C8—C7—C13—C12	175.1 (2)	C16—C15—N3—C12	−179.58 (18)
C6—C7—C13—C12	−0.1 (2)	C14—C15—N3—C12	−0.4 (3)
N4—C14—C15—N3	3.6 (3)	C12—C13—N4—C14	−1.7 (3)
C19—C14—C15—N3	−177.29 (19)	C7—C13—N4—C14	174.96 (18)
N4—C14—C15—C16	−177.21 (19)	C19—C14—N4—C13	178.54 (19)
C19—C14—C15—C16	1.9 (3)	C15—C14—N4—C13	−2.4 (3)
N3—C15—C16—C17	178.0 (2)		

### Hydrogen-bond geometry (Å, º)

Cg1 is the centroid of the C14–C19 ring.

**Table:**

*D*—H···*A*	*D*—H	H···*A*	*D*···*A*	*D*—H···*A*
C41—H41*A*···N3	0.97	2.39	2.988 (3)	120
C11—H11···*Cg*1^i^	0.93	2.89	3.655 (2)	140

Symmetry code: (i) *x*, −*y*, *z*−1/2.
