# Supplementary figures and images for: Crystal structure of 1′,1′′-dimethyl-4′-(4-cholorophen­yl)di­spiro­[11H-indeno[1,2-b]quinoxaline-11,2′-pyrrolidine-3′,3′′-piperidin]-4′′-one
Source: Acta Crystallogr E Crystallogr Commun. 2015 Jan 3;71(Pt 2):o68–9. doi: 10.1107/S2056989014027698 (PMC4384562; doi:10.1107/S2056989014027698)

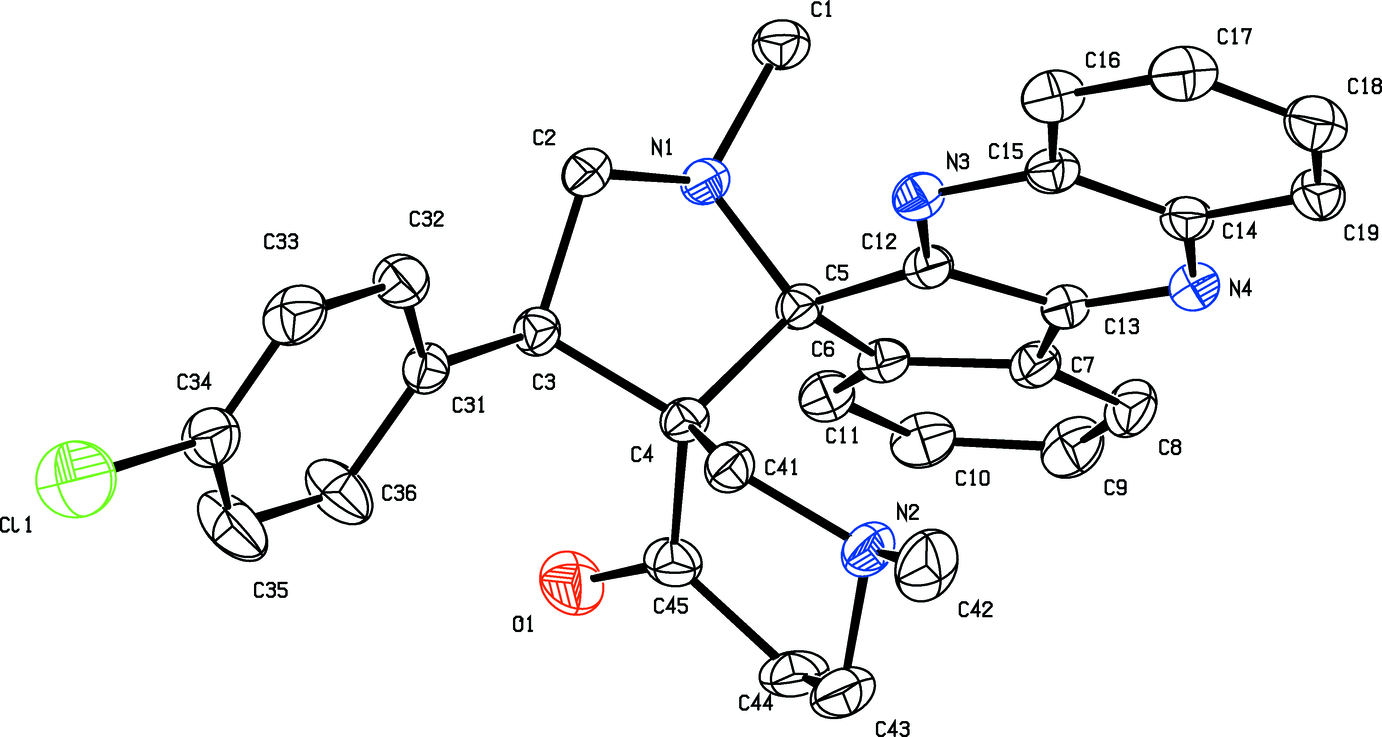

Supplement: Supplementary file 4 [file e-71-00o68-fig1.tif]

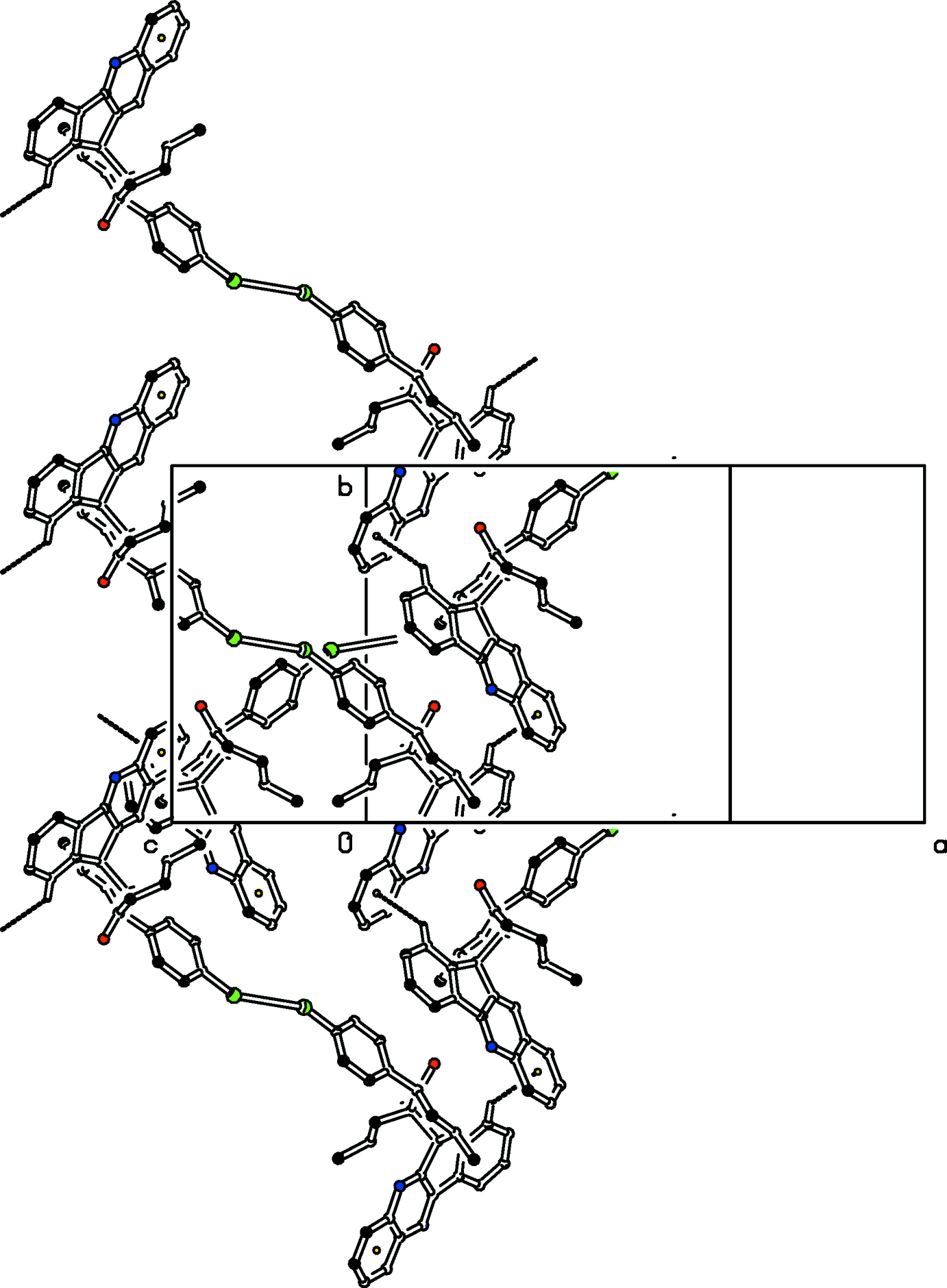

Supplement: Supplementary file 5 [file e-71-00o68-fig2.tif]
